# Proxies of Trustworthiness: A Novel Framework to Support the Performance of Trust in Human Health Research

**DOI:** 10.1007/s11673-024-10335-1

**Published:** 2024-03-29

**Authors:** Kate Harvey, Graeme Laurie

**Affiliations:** School of Law, University of Edinburgh, Edinburgh, Scotland

**Keywords:** Trust, Trustworthiness, Values, Consent, Privacy

## Abstract

Without trust there is no credible human health research (HHR). This article accepts this truism and addresses a crucial question that arises: how can trust continually be promoted in an ever-changing and uncertain HHR environment? The article analyses long-standing mechanisms that are designed to elicit trust—such as consent, anonymization, and transparency—and argues that these are best understood as trust represented by *proxies of trustworthiness*, i.e., regulatory attempts to convey the trustworthiness of the HHR system and/or its actors. Often, such proxies are assumed to operate as markers that trust exists or, at least, has not been lost. But, since trust can neither be “built” nor “secured,” this is a precarious assumption. Worryingly, there is no existing theoretical account of how to understand and evaluate these proxies of trustworthiness as part of a trusted HHR ecosystem. To remedy this, the paper argues for a radical reimagining of trust and trustworthiness as performative acts that ought to be understood in relation to each other and by reference to the common values at stake. It is shown that proxies of trustworthiness are the operational tools used to perform trustworthiness. It advocates for a values-based approach to understanding the relationship between trust and trustworthiness. This establishes a strong basis for an evaluative framework for proxies of trustworthiness, i.e., to determine how to perform trustworthiness *well*. Five common proxies in HHR are scrutinized from a values perspective. The contribution is to provide a far-reaching normative and practical framework by which existing and future proxies of trustworthiness can be identified, assessed, maintained, or replaced in rapidly changing HHR regulatory ecosystems where trust itself is crucial to the success of the entire HHR enterprise.

## Introduction

Public trust in science generally, and in human health research (HHR) more specifically, has always been in the balance (Kerasidou 2017). Most recently, however, a confluence of factors has exacerbated concerns that trust might be in crisis. The issues are myriad and complex and include (i) the COVID-19 pandemic and vaccine hesitancy connected both to expedited approvals (Limaye et al. 2021) and structural injustice (Robertson et al. 2021), (ii) misinformation about, and politicization of, efforts to address the pandemic (Cross 2021), (iii) commercial involvement and suspicion of preferential treatment by regulators in drug development (Karlawish and Grill 2021), (iv) the role of social media in undermining trust (Huber et al. 2019), and (v) the general and constant uncertainty inherent in HHR and how this can be manipulated for political and economic ends (Kreps and Kriner 2020).

Against this background, the core question for this article is: *how can trust continually be promoted in an ever-changing and uncertain environment in HHR?*

The article proceeds as follows. Section 2 examines the relationship between trust and trustworthiness, arguing that a radical re-imaging of each as *performative acts* within the researcher–participant relationship is necessary to establish a sound basis for any normative claims about both features in the HHR ecosystem. Section 3 introduces the notion of *proxies of trustworthiness* to reflect the precarity of what is at stake when trust and trustworthiness are in play. The language of “proxy” captures the idea that attempts to demonstrate trustworthiness might be flawed or might fail to elicit trust. Nonetheless, the argument is made that when proxies of trustworthiness are seen as performative acts oriented towards performances of trust—and relative to the common values that underpin the trustor-trustee relationship—this significantly increases the opportunities for normative alignment in the cooperative exchange between the parties that sits at the heart of HHR. It is shown that proxies of trustworthiness are the operational tools used to perform trustworthiness. Five common proxies to perform trustworthiness in HHR are identified and discussed relative to the fundamental values that they embody. This values perspective reveals strengths and weaknesses of each of the proxies qua performative acts and sets up the analysis in the second half of the article in which a wider values-based framework is developed to provide a grounding for examination and evaluation of all current and future proxies of trustworthiness and their role in seeking to elicit trust. By these means, the article offers a theoretical contribution to understandings of trust and trustworthiness as performative acts as well as a practical approach to promote reflection, action, and legitimacy—from a core values perspective—on acts designed to demonstrate trustworthiness in HHR.

## On the Relationship Between Trust and Trustworthiness

It is a truism that for HHR to thrive there must be trust on the part of participants and the public because, quite simply, without their involvement no ethical or socially acceptable research can take place. Yet, the nature of trust itself remains stubbornly elusive. Trust has been described variously as a belief (Holton 1994) or an affect (Baier 1986) or an attitude (Frost-Arnold 2014), and the literature is replete with attempts at definition (Lahno 2004). It is not the purpose of this article to contribute directly to that ontological debate; rather, we favour the position of Lahno in focusing on the role of trust in bringing about effective social cooperation. In this respect, trust has both instrumental and inherent value in HHR.

Pragmatically, if we receive an offer to participate in HHR, trust plays a key role in how we respond. As part of such an offer, researchers are ethically and legally required to assess the potential benefits and risks of the research and communicate these clearly to potential participants, including how risks will be addressed and minimized (CIOMS 2016). If we trust the research offer (and those making the offer), we may agree to participate because we anticipate those potential benefits (Strömmer et al. 2018) while expecting appropriate safeguards during our participation. However, a faltering of trust might occur when there is a lack of communication between parties, leading to suspicion and distrust of the research offer (Singh and Mills 2005); or where there is no “offer” as such and research proceeds without consent; or, if researchers are found—or perceived—to have a conflict of interest (Wadman 2018); or when there is suspicion of the value of the research, as witnessed with COVID-19 vaccines (Palamenghi et al. 2020). Moreover, mistrust might be prevalent in groups with good historical reason to distrust medical research (Thompson et al. 2021): “social inequality drives mistrust” (Jaiswal and Halkitis 2019, 80; Centre for Disease Control and Prevention 2020). But there is also value in all of this: potential mistrust incentivizes researchers to demonstrate the ethical robustness of their research and any offer that is made. Instrumentally, the prospect of *mistrust* supports the mitigation of jeopardy in HHR.

As to the inherent worth of trust (Candlin and Crichton 2013; McCraw 2015; Carter and Simion 2020; Bok 1978, 31) identified the connection between trust and what we value in our lives: “[w]hatever matters to human beings, trust is the atmosphere in which it thrives.” And, when we ask what matters in HHR, there are myriad answers including the pursuit of a common good, the proper protection of participants, and the realization of social value (Habets et al. 2014; Ganguli Mitra et al. 2017). It is through effective social cooperation between participants, researchers, and research institutions that these matters are made real. For trust to thrive, however, participants must be able to trust that the other parties in this enterprise will deliver. This brings us to the importance of trustworthiness and the central question of its relationship with trust.

Trustworthiness is often defined as the quality of a person or a practice that establishes conditions that have a reasonable prospect of attracting trust (Baier 1986; Potter 2002; Jones 2012). Certainly, this explains a form of relationship between trust and trustworthiness in that it supports an instrumental view of trust that drives an imperative to demonstrate trustworthiness as a character trait. But it is here that we face a conundrum in our attempts to understand fully the relationship at hand. The above definition of trustworthiness only provides a “thin” version of the concept, i.e., it offers necessary but not sufficient criteria to actually *be* trustworthy. The classic con person will be adept at signalling conditions to attract trust but their intention all along will be to deceive and so betray any trust. A “rich” conceptualization of trustworthiness would need to look into, say, underlying virtues, intentions, and dispositions (Potter 2002), and we return to aspects of this below.

Furthermore, the instrumentalization of trust and trustworthiness can lead us to overlook the inherent values at stake in trust and trustworthiness, including values such as the advancing of authentic human relationships, securing genuine respect for persons, promoting dignity and integrity, and furthering accountability (Provis 2001). Finally, to see trustworthiness as an inherent trait of persons or institutions, and trust as something that is done or given, sets up a problematic dynamic between these two concepts and presents real hurdles to understanding them—and their relationship to each other—in meaningful ways. This is because the dynamic involves conceptualizations that are inherently incommensurable with each other.

Carter (2023) has recently offered a way through this impasse by suggesting that we can establish common normative ground by examining each concept through a performative lens. Thus, rather than seeing trust as “doing,” i.e., giving trust, and trustworthiness as “being,” i.e., having an attribute, when both are seen as performative acts it becomes normatively more meaningful as a basis both to understand such acts relative to each other and as a means to develop a normative evaluative framework of the roles of trust and trustworthiness in “successful cooperative exchanges” (Carter 2022). “Performance” here should be understood as “. . . any state or action or process that has a constitutive aim” (Sosa 2015 chapter 5, FN5; Sosa 2021). Any human action is a performance if it is directed towards a particular outcome. Thus, the cellist performs with the aim to entertain their audience, and the golfer performs with the aim, ideally, to hit a hole in one. Inherent in this definition are the notions of success (and its corollary, failure) and competence. Applied to the HHR ecosystem, success is the realization of social value from research but, in turn, this is highly contingent on the means used to achieve such an aim. It is not social value at any cost, because the ethics and governance of research matter very much, including assessments of the competence of researchers to deliver. Indeed, the success of HHR is the *product* of an effective cooperative exchange between participants who trust (trustors) and researchers and institutions (trustees) who must deliver and whose competence to do so, i.e., trustworthiness, is crucial. But what, then, are the respective performances of trust and trustworthiness associated with this idea of success?

For participants, the performance of trust is the act of trusting itself. In HHR, successful trust occurs not merely when the social value is realized but when this occurs *as entrusted*, e.g., as promised in the research offer and/or with due protection. For researchers and institutions, trustworthiness can be said to be successfully performed also when the research aim is delivered *as entrusted*. This will require a disposition to act positively towards the trustor and their expectations *as manifested through their performance of trust*. In this way a performance of trust and a performance of trustworthiness can be understood relative to each other and can also be evaluated relative to a shared sense of what counts as success. The implications of this for HHR are explored fully in the next sub-section.

This performative framing of trust and trustworthiness provides a basis to understand and evaluate any successful cooperative exchange in HHR. It can be contrasted starkly with most existing accounts of trustworthiness which focus on the disposition of the trustee and which might have nothing to do with the act of trusting. For example, someone might be entirely trustworthy as a person and yet not elicit trust; contrariwise, trust might be misplaced in someone whose disposition is oriented towards perpetuating a fraud (O’Neill 2018). However, through the performative lens there is a greater potential for normative alignment. As Carter (2023, 382) explains:

(T)he trustor (through trusting) aims not just at the trustee merely being a certain way—or even at the trustee doing a certain thing while at the same time being a certain way—but at the trustee achieving a certain thing, viz., succeeding in taking care of things through their trustworthiness.

This provides a robust basis on which to discuss and evaluate the roles of trust and trustworthiness as aspects of a (successful) cooperative exchange in ways that are not exclusively focused on what the parties are doing or the dispositions they have.

Sosa (2015, 2021), Fernandez Vargas (2016), and others (Kelp et al 2020) have examined elsewhere the nature of human performance and its normative evaluation, offering and critiquing criteria for what successful performance looks like. When applied to trust and trustworthiness as performances, this can be valuable to help judge when trust is well placed or misplaced, i.e., whether the performance is successful and apt (Carter 2021), but that is not the particular exercise that concerns us here (Carter, “Trust as Performance,” 2022). Rather, by presenting both the act of trusting and the act of seeking to demonstrate trustworthiness as *performances* oriented towards a common goal, we simultaneously provide a means to talk about trust and trustworthiness as being ontologically of the same kind and direct attention to the values at the heart of HHR that are common to the concerns of all stakeholders, trustors and trustees alike. This becomes important for any normative evaluative assessment of performances of trust and trustworthiness. The implications of such a values-based approach are explored fully below. For now, we must ask: what does a performative turn imply for HHR and its actors?

### Performing Trust and Trustworthiness in Human Health Research

To appreciate the implications of a performative turn for trust and trustworthiness in HHR, it is important to briefly consider the character of the HHR ecosystem found in most countries that follow international ethical standards (World Medical Association 2013; CIOMS 2016). This reveals where issues of trust (or mistrust) might arise and allows us to identify where research actors might be called upon to perform trustworthiness.

**Figure 1:**
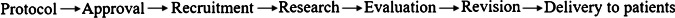
HHR: Constituent elements

We posit that the HHR ecosystem is best viewed as a trajectory rather than a single entity “event,” as represented by figure 1. This trajectory is comprised of a series of points of significant human interaction with feedback loops where learning and instances of good/bad practice reflect back on the system and can positively or negatively impact the course of research (Taylor-Alexander et al. 2016). Figure 1 should not be read to suggest that HHR is a single linear process. Rather, the schematic reveals junctures that represent “moments” in the trajectory where performances of trustworthiness are particularly important and where concerns for trust/mistrust will be especially acute.

Completion of the trajectory will only result when there has been a series of successful cooperative exchanges between researchers and participants at all points of interaction.

Broadly, cooperative exchange in HHR has meaning at multiple instances for the different actors. For example, when participants perform trust at the recruitment stage, they give something of themselves (e.g., their data, tissue, and/or time). They entrust that scientifically sound, ethically robust research will be undertaken with the reasonable prospect that (public) health benefits will result and social value will be realized. More particularly, the act of entrusting that the research is ethical includes the expectation that core values such as respect for participants, privacy protection, and harm minimization will be honoured and that the research will be conducted by competent professionals. Contrariwise, sometimes the performative element of trust is achieved through inaction. An example is data-driven research that offers an opt-out. By not exercizing an opt-out, citizens “perform” an act of trust in the research endeavour.

Equally, reciprocity of trust is in play: researchers trust that participants take the research process seriously and will contribute as required. The cooperative exchange here is the delivery of robust research results with due respect and care.

This leads us to consider the performativity of trustworthiness. What does it mean for a research actor or institution to perform trustworthiness successfully, i.e., as entrusted? This will take different forms at different junctures of the HHR trajectory. For example, for research involving informed consent, the terms of exchange are constituted by the consent form. To perform trustworthiness successfully is to behave as promised in the original consent. But even this seemingly straightforward alignment of trust and trustworthiness suffers from immediate precarity. Consent is neither formally contractual nor exhaustive in its terms (Laurie and Postan 2013). Research protocols are subject to change as research unfolds, and it is not always feasible or possible to return to participants to update consent. In such cases, the ongoing performance of trustworthiness must be done by other means, e.g., through renewed ethical review and adherence to new recommendations from the ethics body, e.g., a research ethics board or institutional review board. But a dilemma then arises because there is no longer a demonstrable connection between the original trust as performed through the signing of the consent form and any future attempt to perform trustworthiness. That is, despite best efforts to act ethically, there might no longer be an alignment between performances of trustworthiness and performances of trust because the researchers have not performed trustworthiness *as entrusted*. This is not necessarily fatal to trust or to the research endeavour in any given project, but it does raise a deeper problem that exists across the entire HHR research trajectory and for all forms of research.

The problem is this: all attempts to perform trustworthiness potentially fail because of the risk of a disconnect between trust and trustworthiness—i.e., the trustee simply does not know whether attempts to perform trustworthiness necessarily or accurately reflect the expectations that underpin any trust that is given. It is for this reason that it is crucial to recognize that trust is not something that can be simply “built” despite so much rhetoric among stakeholders to this effect (Academy of Medical Sciences 2011). Research actors along the research trajectory can work hard to demonstrate their own trustworthiness and to act in ways that provide good reasons for people to give their trust, but they cannot assume that this will attract trust. Nor can they rely on their own good conduct to construct trust because this is something that comes from another party over whom there is no direct control.

The problem is compounded because trust is precarious both at the individual project level and at the systemic level. As the above example involving consent demonstrates, research is inherently uncertain (Heneghan et al. 2017). At the project level, this means that trust remains under constant threat from the prospect of constant change within a research protocol. Furthermore, trust is precarious because of systemic tensions between different objectives of the research ecosystem. For example, in one direction is a pull towards regulation that delivers robust standards of protection, the paradigm example of which is the clinical trial and the securing of informed consent (Rosemann 2019). In the other direction is a push towards the promotion of innovation in the face of enduring uncertainty; this might involve obligations on researchers to make their research findings and data more “open” to the scientific community, but with attendant risks to citizens’ privacy rights (Royal Society 2012).

Threats to trust are further exacerbated as biomedical research becomes increasingly complex and further removed from the classic researcher-participant relationship based on explicit consent. For example, big data research involving anonymized datasets usually does not proceed on the basis of consent, and the ethics and legality of this have been examined elsewhere (Callréus 2022). This kind of research involves a growing range of actors whose trustworthiness then also becomes important, e.g., data custodians and data access committees. But how are they to perform trustworthiness well? Equally, what does it mean for participants to trust in such research when the research actors might be entirely unknown?

The suggestion that we reimagine trust and trustworthiness as performative acts relative to each other does not answer these questions. It does, however, reveal the nature and the depth of the problem that we face: the health research ecosystem sets up a dynamic that is built on a precious and precarious form of social cooperation that has trust at its core, but those seeking to perform trustworthiness well are often in the dark about what to do to honour trust. But some hope is to hand because understanding the relationship between trust and trustworthiness in performative terms at least provides a means to ally them together in ways that are meaningful for the respective parties who seek a successful cooperative exchange. Indeed, an ideal emerges to strive for wherein trust and trustworthiness can be aligned *as entrusted*. This in turn allows us to say something normative about trustworthiness: a performance of trustworthiness is truly effective, i.e., successful, when it achieves the common goal as entrusted and with a disposition that is oriented towards the expectations of the trustor. But the stubborn problem in HHR remains: it is rarely possible to know with confidence the basis upon which trust itself is performed, and this returns us to the central research question of this article: how can trust continually be promoted in an ever-changing and uncertain environment in HHR?

We suggest that the answer lies in a reorientation of our understandings of health research regulation on the basis of this performative turn for trust and trustworthiness and in two key respects: 1. to conceive the actions of researchers and research institutions as mere *proxies of trustworthiness* in recognition of the fragile relationship to trust, and 2. to establish an ethical framework to evaluate those proxies of trustworthiness relative to the core values at stake. As a first step towards answering our central research question, we argue in the next section that all performances by research actors ought to be understood as proxies of trustworthiness.

## Proxies of Trustworthiness

Human health research is replete with regulatory mechanisms designed to protect participants and promote ethically sound research. Potential research participants are presented with a bewildering array of “good reasons” to trust the research enterprise. Examples range from consent mechanisms to robust data anonymization practices; from rigorous ethics review to opportunities for engagement in the research trajectory at various junctures; from transparency of protocols and research practices to accountability of research actors and institutions in the short and longer term. Each of these mechanisms represents an occasion for research stakeholders to perform trustworthiness. But, as demonstrated above, the fundamental problem remains that we cannot know whether any given performance of trustworthiness is necessarily allied to performances of trust.

To capture the precarity of this relationship and to better inform action-guiding decision-making in HHR, we suggest that it is helpful to talk in terms of proxies of trustworthiness. Each of the above regulatory mechanisms should be styled as such. A “proxy” embodies the notion of “as if”—just as customer loyalty serves as a proxy for customer satisfaction, so too research participant retention might be taken as a proxy for participant trust. And the analogy applies equally to trustworthiness: just as fulfilment of a contract serves as a proxy for business reliability, so too research actor performances done through robust regulatory mechanisms might be taken as proxies of trustworthiness. Thus, research actors’ deployment of mechanisms in their research such as consent and/or anonymization and/or public engagement strongly signals that they are acting, i.e., performing, *as if* they are trustworthy. This framing in no way is to cast aspersion on these actors. Rather, to talk of a proxy of trustworthiness is to acknowledge the unknowns on both sides, i.e., from the trustee’s perspective there is the unknown about expectations of the trustor, and from the trustor’s perspective there will often be unknowns about motives or influences underlying a trustee’s actions. On the proposed framing, the focus is on what is actually done—i.e., performed. Proxies of trustworthiness are thus the operational tools used to perform trustworthiness. This is different from any assessment about whether trustworthiness is performed well. So, how can such proxies be evaluated as to their effectiveness and relative to any performances of trust?

It has been suggested above that the ideal is when performances of trustworthiness align with performances of trust *as entrusted*. But like most ideals, this situation will remain largely elusive. Contrariwise, performances of trust might be for highly subjective reasons that bear no reflection on the performativity of trustworthiness of the research actors, e.g., a person signs up for research out of loyalty to their deceased parent’s wishes. This would represent the furthest point from alignment of trust and trustworthiness. But, as with the ideal, this is likely to reflect only a tiny minority of situations. Rather, it is reasonable to assume that most performances of trust will be directed to a material extent towards trusting the system to deliver health research outcomes and to uphold the foundational values that support the operation of the entire research enterprise. Accordingly, if there is a demonstrable commitment to the same values manifested through proxies by those seeking to perform trustworthiness, then this will greatly increase the chances of normative alignment between performances of trustworthiness and trust.

This provides a starting point for research actors to reflect on how to increase the chances of a successful cooperative exchange in HHR. The challenge becomes how to deploy proxies—alone or in combination—to perform trustworthiness *well* and in a way that will best align with trust at any given juncture in the research trajectory.

Minimally, proxies of trustworthiness ought to reflect (some) common shared values within the ecosystem. This is the source of their legitimacy qua any claim about trustworthiness. Maximally, when performed relative to performances of trust, the two will align, i.e., trustworthiness will be manifested as entrusted through the performance of trust and relative to values shared by all parties. But given the dynamic nature of the research trajectory as represented by figure 1, it is important to recognize that the search for alignment will be an ongoing process. This is especially true if a research protocol is changed or if a research scandal arises. It does not follow, however, that in such cases trust is automatically misplaced nor that trustworthiness cannot be adequately performed. As suggested above, a change in protocol can be quite common and might require a change in proxies of trustworthiness. For example, longitudinal research based on consent might not initially foresee the significance of downstream research findings nor how to deal with them. Thus, the original proxy of trustworthiness—consent—will not assist with new downstream issues. But the prospect of a renewed ethics review of the protocol—ensuring due reflection on the emergent issues—can serve as a new proxy through which trustworthiness can continue to be performed (albeit that alignment may then be suboptimal relative to the original consent). In a research scandal, a participant might continue to perform trust even if some actors have performed in an untrustworthy fashion. Is such trust misplaced? How can we tell? Well, if it is about continuing to trust actors whose performances were deceitful, then the trust can be adjudged to be misplaced because the central values of honesty and integrity have not been honoured. It is common ground that these values are fundamental to a robust HHR system (Xafis et al. 2019). In contrast, if the performance of trust is oriented towards the system itself, and if that system and its values root out untrustworthy performances and hold the actors accountable, then that trust is arguably not misplaced. Bad apples do not necessarily rot the barrel.

It can be seen, then, that the common values of the HHR ecosystem ground both performances of trust and trustworthiness. An assessment of the degree of alignment must begin with those values. And an account of those values within particular proxies of trustworthiness therefore becomes crucial to any such exercise.

### Five Common Proxies of Trustworthiness

An examination of five commonly deployed proxies of trustworthiness explains attempts to perform trustworthiness relative to underpinning values. These are: (i) consent, (ii) anonymization, (iii) public engagement, (iv) openness, and (v) accountability. Other candidate proxies could include attempts to minimize risks, ensure community benefit, treat subjects fairly, extend diversity of participation, and/or promote valuable science, among others. There is no normative preference in the current selection, nor is it suggested that these enjoy particular privilege or success. Rather, together they form a character profile of trust-seeking behaviour and allow us to explore what is happening at the heart of these proxies when stakeholders rely on them to perform trustworthiness. In this section, the five candidates are explored briefly, with a focus on how each might represent a proxy of trustworthiness: this question is answered by making explicit the values that each proxy elicits. It is these values that make the examples compelling candidates as proxies of trustworthiness.

- ***(i)*** *Informed Consent as a Proxy of Trustworthiness*

The role of consent in health research has been discussed above. The classic research paradigm, as typified by the clinical trial, privileges consent in that people with appropriate capacity should not normally participate in HHR unless they have given their informed consent (World Medical Association 2013, 25–32). This principle is clear in guidance and regulation and in international research practices (FDA 2023; CIOMS 2016). To perform trustworthiness, researchers must clearly communicate what they are offering, and in what form, so that potential participants can respond to that offer. Continued performance according to the conditions of the consent aligns trust and trustworthiness in performance terms, perhaps optimally so in the first instance.

#### Which values are elicited through this proxy of trustworthiness?

Participants trust that researchers are clear and honest in how the offer is presented, including relevant risks. Researchers trust that potential participants engage seriously with the offer. Each party is expected to be respectful of others’ roles. The values in play are therefore autonomy, respect for persons, and honesty as an assurance that deception and coercion are absent (O’Neill 2003). Such assurance makes informed consent a prime candidate as a proxy of trustworthiness. If consent is given, it is a prima facie indicator that trust is present (at the time when consent is given). However, the strength of consent as a performance of trust might diminish along the research trajectory as factors in the research change, and as highlighted above. This is a limitation of consent as an ethical protective practice, and this is why consent must be cast as a proxy: its durability cannot be assumed. Put otherwise, a signed consent form does not establish that researchers or their research have adequately performed trustworthiness across the entire research trajectory, nor that the initial performance of trust can be relied on with impunity.

- ***(ii)***  *Anonymization as a Proxy of Trustworthiness*

When personal data are gathered for HHR, there is an expectation that research actors will not disclose data to others without prior agreement or good reason (HRA 2020a). Upholding confidentiality therefore commonly forms part of the HHR “offer” to participants, often operationalized through anonymization of participants’ data (EMA 2017). Thus, the performance of trust can rely heavily on the undertaking that anonymization and confidentiality will be maintained. Indeed, this is also true when there is no research “offer” as such, because anonymization is frequently used when consent cannot be relied upon, i.e., a common approach in HHR is the “consent or anonymize” model (AMS 2011; Dove and Laurie 2015).

The character of anonymization as a proxy of trustworthiness reflects its precarious nature. First, it is merely a technical measure concerned with risk minimization; as such, there is no guarantee of success and risks are multiplied with extended data use (AMS 2006). Second, appeals to public interest to disclose information might sometimes trump participant confidentiality (Taylor 2015). In such cases, decisions to override participants’ expectations also need to consider the public interest in maintaining overall trust in a confidential healthcare system or a well-functioning HHR ecosystem (Taylor and Wilson 2019).

#### Which values are elicited through this proxy of trustworthiness?

Privacy is the core value promoted by anonymization. We cannot assume, however, that people value privacy to the same (high) degree; privacy is a subjective notion and lacks constancy (Sethi and Laurie 2013). Moreover, privacy tradeoffs are common. But assuming privacy is valued to some degree, many individuals will make decisions to participate in research based on assurances to respect confidentiality and to deploy anonymization: participants trust their personal details will not be “noised abroad.” Anonymization also manifests the value of integrity. Integrity is a demonstrated key value to maintaining trust in HHR (Coughlin et al. 2012).

Inherent in this is an enduring challenge: just because a person expresses a privacy preference does not guarantee their preference will be met, particularly if competing interests are in play (Coughlin et al. 2012). Thus, there is a constant risk that performances of trust (based on expectations of privacy) will not align with performances of trustworthiness (done through anonymization and confidentiality). For these reasons, confidentiality and anonymization should be cast as proxies of trustworthiness.

- ***(iii)*** *Public Engagement as a Proxy of Trustworthiness*

There is now a well-established expectation that researchers engage with those whom their research affects (NIHR 2019; HRA 2020a, b). With respect to trust, public engagement (PE) can generate evidence of what publics will accept, tolerate, or reject; that is, what they will or will not trust. However, the limitations of all PE exercises mean that the evidence base is partial at best and never fully representative. Equally, PE also operates to support other proxies by providing an evidence base for their use (or rejection).

#### Which values are elicited through this proxy of trustworthiness?

The value of PE rests in its mutual regard and respect for participants (Pieper and Thomson 2014), provided this is not tokenistic (Hahn et al. 2017). A commitment to robust PE is, therefore, a strong proxy of trustworthiness because it seeks evidence about how better to align performances of trustworthiness with performances of trust. Indeed, when the results of a PE exercise involving participants are transposed effectively into the conduct of research itself, there is an increased likelihood of alignment of performances of trust and trustworthiness. The language of proxy remains pertinent, however, because we do not always get what we (think) we want; the plurality of views invariably revealed by PE means that, necessarily, some voices will be preferred over others.

- ***(iv)*** *Openness as a Proxy of Trustworthiness*

Openness in HHR is recommended in guidance and regulation (World Medical Association 2013, para 35) and through policies that urge datasets to be open and available (Royal Society 2012). Openness operates as a proxy of trustworthiness in ways similar to robust PE: it demonstrates researchers are open to discussing their data and findings with other researchers and the public and perhaps changing conduct in light of discussion (UKRI 2022). Openness is a proxy of trustworthiness through researchers’ demonstration that they have nothing to hide, that they believe in the quality of their research and findings, and that they share a common goal of the advancement of science.

Although openness and transparency might be used synonymously as features of HHR that support claims of trustworthiness, they are different. Openness is characterized by accessibility as well as assessibility (e.g., as to the value of research data). Transparency makes information available publicly without doing the facilitative work necessary to allow non-researchers to engage with it meaningfully (O’Neill, British Science Association 2016).

Openness as a proxy of trustworthiness can be observed in specific HHR contexts, including where researchers provide feedback on research results to participants (Moreno-John et al. 2004). Not only is follow-up supported by regulators (HRA 2015), but some participants also welcome feedback because it exemplifies reciprocity (Ralefala et al. 2020). This is an illustration of an ongoing performance of openness and demonstrates how downstream performances of trustworthiness can be very important in the HHR trajectory.

#### Which values are elicited through this proxy of trustworthiness?

Openness elicits notions of exchange and reciprocity. It supports cooperative exchange among all stakeholders. It also engenders respect by moving away from HHR being a closed shop to enabling non-researchers’ (including participants’) engagement with information that supports a frank account of the HHR. Yet, despite commitments to openness, misinformation or deception may still operate in HHR (O’Neill 2002). Recognizing such limitations again supports the proposition that openness is at best a proxy of trustworthiness. As with the other proxies discussed so far, openness is part of a network of mechanisms that supports claims about a trustworthy HHR ecosystem.

- ***(v)*** *Accountability as a Proxy of Trustworthiness*

In assessing any research ecosystem, mechanisms of accountability are strong indicators of trustworthiness because they require compliance with regulations and standards (O’Neill 2004). Accountability means research actors are responsible for their actions and must be prepared to justify themselves to those to whom they are accountable in a legal sense (e.g., through regulatory compliance), as well as to those affected by their acts or omissions in an ethical sense.

Accountability mechanisms support the performance of trustworthiness by reassuring participants that, if anything goes wrong, there are consequences. These ensure responsibility is taken and that checks are in place to mitigate harm (Kass et al. 1996) and uphold research integrity (Guillemin et al. 2018). Accountability mechanisms are, therefore, proxies of trustworthiness because their existence might encourage participants to take part when, without such reassurances, they would not do so. A strong system of accountability is a performance of trustworthiness by the system itself.

#### Which values are elicited through this proxy of trustworthiness?

Accountability offers potential participants reassurance that the system is fair and just. It also indicates an underpinning value of respect through showing the system offers care for participants.

### The Value(s) of Proxies of Trustworthiness

It is important to recall the dynamic nature of HHR, an indicated above. As it shifts, so too will the effectiveness of its proxies of trustworthiness, and the relative weight afforded to any proxy. For example, while consent might be the dominant paradigm in clinical trials, it is less effective (and often impracticable) in data-driven innovation where anonymization comes to the fore (Kalkman et al. 2019). While anonymization mitigates some privacy risks in many types of research, its utility is diminished in the era of big data when datasets from multiple sectors and ecosystems come together. This means that robust PE and clear lines of accountability become more important (Xafis et al. 2019). Sometimes a mechanism might endure for the whole research trajectory, e.g., reliance on informed consent. At other times, a proxy might only apply at a particular point, e.g., public engagement prior to or during recruitment. Moreover, because proxies of trustworthiness are battling a shifting environment, they also leave the ecosystem vulnerable to its weakest element: if one proxy of trustworthiness is not well executed it can severely affect the entire enterprise. In sum, there is a persistent need to perform ongoing trustworthiness and to recognize that proxies do not sit in isolation from each other. Rather, they form a network that can be drawn upon when research actors seek to align their performance(s) of trustworthiness with performances of trust. Where one proxy is found lacking, another may take up its mantle. There is no magic formula to deploy proxies of trustworthiness to secure performances of trust and far less to align performances of trust with performances of trustworthiness across the entire research trajectory.

By understanding the pursuit of trust as represented by a collection of interwoven proxies of trustworthiness, the HHR system and its research actors are required to stay alert. This means the system avoids the hubristic assumption that trustworthiness in HHR is something that can be secured permanently. This cautious approach is necessary precisely because trust itself is fragile and fickle. Thus, a proxy of trustworthiness introduced to elicit trust in a HHR scenario might fail when applied. Constant reflection on these proxies is needed because, as circumstances change, so might their efficacy. And, while it is facile to declare fixed matrices are inappropriate, it is more challenging to suggest which proxies might be used in a given context when research actors are seeking to perform trustworthiness relative to performances of trust.

The identification of underlying values is an attempt to rise to this challenge. As indicated above, this approach is defensible because the core values represent a meta proxy of what all actors inherently care about when they engage in HHR. Proxies of trustworthiness are each the embodiment of one or more core values. By making these values explicit, performances of trustworthiness and trust can be better aligned and so adjudged. When the proxies of trustworthiness are viewed as potential courses of action open to stakeholders in the HHR ecosystem, then a discussion of shared values provides a strong normative basis to support their use—or non-use—in any given context.

It would be misguided and self-defeating, however, to assume that the five proxies suggested here are definitive means by which to perform trustworthiness successfully, alone or in any possible combination, and in ways that align with performances of trust. Other proxies might emerge that do a better job for the task of performing trustworthiness well. Rather, by considering all potential performances of trustworthiness as proxy manifestations of underlying values, we have an opportunity to recognize the fragility of trust and give stakeholders a fighting chance to perform trustworthiness well in an ever-shifting HHR ecosystem—so long as they engage fully and robustly with the values at stake. What is lacking, however, is a schema linking abstract values with concrete actions, i.e., proxies. To remedy this, in the next section we offer a framework to support research actors to identify values and reflect meaningfully on why particular proxies of trustworthiness are likely to lead to a performance of trust in a given context. Values are the engine in the proxy of trustworthiness machine. To make the machine work, we need to identify which values come into prominence depending on the proxy in question, recognize where values are in jeopardy, and determine what might be needed to resolve this jeopardy—e.g., if a new proxy is needed, or if an additional established proxy needs to be called into action.

## A Values-Based Framework to Assess Proxies of Trustworthiness in HHR

Using five exemplar proxies of trustworthiness, we have suggested that a values-based approach is useful in understanding the operations of the HHR ecosystem. What remains elusive, however, is a means to evaluate and assess the worth of these proxies of trustworthiness, especially when it cannot be guaranteed that they will indeed elicit trust. Moreover, we must contemplate that with new developments and threats in the HHR ecosystem, new proxies of trustworthiness might be required. For example, the conduct of HHR during a public health emergency where time is of the essence might mean that “conventional” approaches are not fit-for-purpose; in such cases, researchers and other decision-makers need clear guidance on how to think through the ethical issues at stake and decide how best to proceed (Nuffield Council 2020) and how best to communicate with publics (Lowe et al. 2022). In what follows, a values-based *framework* is proposed to assist in these tasks.

### Why is a Values-Based Framework Appropriate?

Frameworks can help us identify, methodically, issues at stake in a particular context (Xafis et al. 2019). They are practical tools to consider complex scenarios and—importantly—do not prescribe a particular “answer” but instead offer a flexible, pragmatic approach that promotes action (ter Meulen 2016). Examples of successful frameworks to date include Dawson’s work on public health ethics (Dawson 2010) and the SHAPES Working Group on Ethical Decision-Making in Big Data (Xafis et al. 2019).

But why do we need a *values*-based framework? It has been argued that proxies of trustworthiness are the operational tools used to perform trustworthiness. However, each of these tools is a proxy of trustworthiness *precisely because* there is no guarantee that the particular mechanism—be it consent, anonymization, public engagement, etc.—will necessarily elicit trust, i.e., that the performances of trustworthiness and trust will align. However, the values underpinning any given proxy of trustworthiness will remain constant. These values are also likely to underpin performances of trust. Thus, in seeking to align trust and trustworthiness, a focus on aligning *values* holds considerable promise.

The identification of underlying values also answers the question—what does it mean to *perform* trustworthiness? In essence, to perform trustworthiness is to give effect to the values at stake in any given proxy (cf. Potter 2002 and Kelly 2018 who advocate a virtues-based approach to understanding and demonstrating trustworthiness). The performance of trustworthiness will be optimal when there is alignment of values as entrusted through a performance of trust. Suboptimal performances occur when there is misalignment of values, and the degree of misalignment is likely to be proportional to the degree of resulting mistrust.

Thus, we contend that what is required is a framework to guide actors to identify key values when attempting to perform their trustworthiness through existing proxies (or through new ones if the extant collection is found wanting). By making these values explicit, the aim is that those proxies, i.e., operational tools, are strengthened. Overall, this improves the chances of creating a trustworthy HHR ecosystem (Lowe et al. 2022). Moreover, scrutiny from a values-basis can reveal both limitations in existing proxies of trustworthiness and the basis on which to establish and put into operation new ones when needed. An open, values-based framework supports attempts to deliver trustworthiness *well*.

#### A Proposal for a Values-Based Framework

Values are an expression of what matters morally in a particular community. Values are commonly shared among community members, or if they are not then good reasons must be given to defend them as action-guiding norms. From the five proxies already discussed and looking more holistically at the HHR ecosystem, the following values can be identified as most relevant for the present discussion. These are grouped into *substantive values*, i.e., considerations that should be realized through the outcome of a decision, and *procedural values*, i.e., values that guide the decision-making process itself.^1^

**Table:** 

*Substantive values*	*Procedural values*
Autonomy	Fairness
Respect for persons	Accountability
Privacy	Transparency
Harm minimization	Integrity
Respect for cultural diversity	Humility
Promotion of valuable science	Explicability
Care	Engagement
Solidarity	Accessibility
Benefit sharing	Affirmative access

This is a non-exhaustive list. Decision-makers might reasonably make a case that other values are in play in each given context. Equally, it must be recognized that some values might create tensions, e.g., protecting privacy to a high level might mean that certain kinds of valuable science using patient data cannot be conducted. No framework for ethical decision making can resolve all possible tensions. Rather, it is for stakeholders to make the case as to which value or values should be prioritized and to defend this robustly. Our framework provides a scaffold for decision-makers to approach this task, placing trust and trustworthiness at its centre.

**Figure 2:**
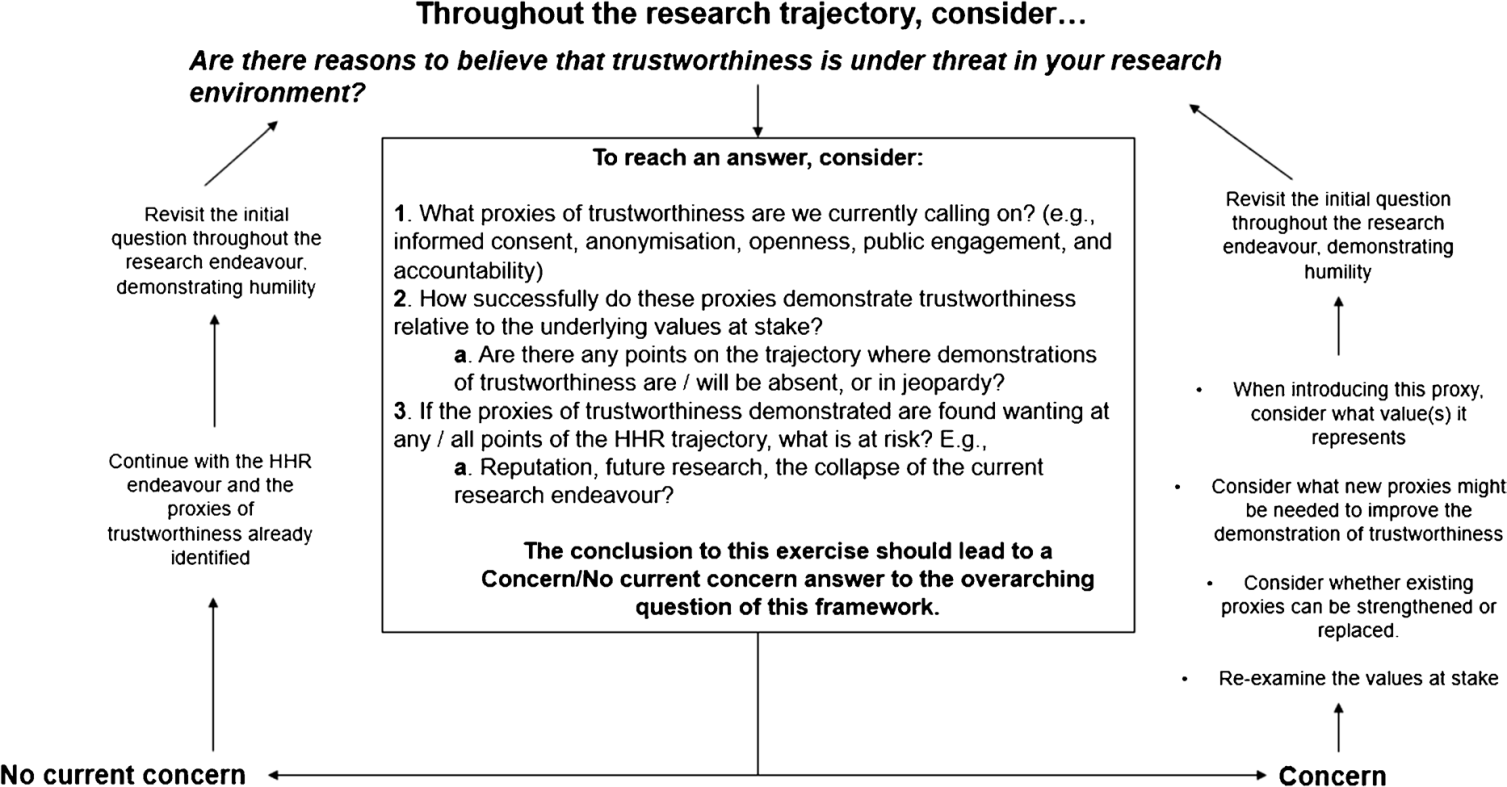
The trustworthiness framework

Figure 2 sets out a proposal for a values-based framework that research actors and their institutions can use to address a pivotal question: *are there reasons to believe that trustworthiness is under threat in your research environment?* The reflexivity of its approach is designed to strengthen performances of trustworthiness in HHR. It also serves as a basis to evaluate any proxies of trustworthiness in play and to establish a basis for new ones, where needed.

### Operationalization of the Values-Based Framework for Proxies of Trustworthiness

This framework should not be deployed only at single points on the research trajectory, but rather *throughout the HHR trajectory* as illustrated by figure 1 above. At each juncture where we ask the questions set out in the framework, we may get different answers because the context is crucial. The framework therefore represents an example of a feedback loop: a cycle of consideration, analysis, and improvement. As the framework is used more frequently, an understanding of how trustworthiness can be performed well across the HHR trajectory will be strengthened by considering extant proxies, and any further proxies that might need to be introduced. The aspiration is that this framework will support a learning, intelligent system where proxies of trustworthiness are analysed for each HHR context and relative to any evidence about the values informing performances of trust (Laurie 2021).

Second, although the framework’s steps can be an individual exercise for research actors, it must be recognized that the HHR ecosystem is comprised of many actors with different roles. What one actor recognizes as a strong proxy of trustworthiness might be understood as weak by another. For example, a clinician-researcher might put a lot of faith in consent, while a data scientist-researcher might prefer anonymization because consent is seen as impracticable. Thus, a whole system approach is required for the framework’s application. That is, all actors with an interest in performing their trustworthiness should apply the framework to their activities. Indeed, they might usefully ask: *are my actions the weakest link in the ecosystem of proxies of trustworthiness?* The expectation is that through reflective equilibrium of the entire ecosystem, the underperformance of proxies—or indeed their successes—are less likely to be missed. But with this comes an important caveat: if any actor or set of actors does not participate fully in this reflective exercise, there is a risk that the whole enterprise is undermined. Put otherwise, the trustworthiness of the entire system is in doubt.

#### The Initial Question in Three Parts

The question at the heart of this framework—*are there reasons to believe that trustworthiness is under threat in your research environment?—*prompts us to reflect critically on efforts to perform trustworthiness well. There are three parts to consider when answering this question.

- *1. The proxies of trustworthiness currently called upon*

This part of the framework encourages actors to identify their performances of trustworthiness via their current reliance on one or more proxies of trustworthiness. It requires, in particular, an account of which values are being promoted (or ignored) via the proxies of trustworthiness. This will also reveal potential tensions. For example, is a desire to protect privacy unduly hindering sound science (or vice versa)? Importantly, the values that likely underpin any performances of trust should also be identified at this point. By these means, the full gamut of values in play will be revealed. From here, an assessment of alignment of values as between performances of trust and trustworthiness can proceed.

- *2. The success of proxies in performing trustworthiness relative to values*

This part of the framework moves from audit to analysis. It prompts research actors to examine how far and how well the proxies of trustworthiness upon which they rely reflect the underlying values at stake, including values that might have come to prominence because of changes in the research ecosystem or wider society.

The framework suggests two prompts to kickstart analysis here. The first prompt asks research actors to consider if there are points on the HHR trajectory where performances of trustworthiness will be absent or in jeopardy. This urges actors to analyse trustworthiness across each part of the HHR trajectory, rather than to undertake analysis as an isolated event. For example, do completely unexpected research findings that formed no part of the consent process now require a re-consent and/or wider engagement with participants? If reconsent is not possible, how will trustworthiness continue to be performed well?

The second prompt asks actors to identify and explicitly reflect on the values underpinning extant proxies of trustworthiness. If there are reasons to question whether the proxies are working well, then this might also mean that the core values shoring up the research might also be open to question. For example, has a reliance on anonymization and privacy protection (at the expense of seeking consent to demonstrate respect for persons) led to mistrust? Similarly, has an early and narrow programme of public engagement led to a failure to respect cultural diversity in the downstream conduct of the research?

Manifestly, this analysis will also have an empirical element, especially regarding trust, i.e., is there tangible evidence that trust is present or under threat? Where evidence is lacking, empirical studies could garner evidence about levels of trust, and this should be done with a view to revealing what participants actually value through their continued participation. But even in the absence of evidence, this element of the framework promotes reflection of how well any particular proxy is operating across the entire research trajectory, e.g., has an informed consent given many years previously now run its course and/or been superseded by new considerations? Might a different proxy, such as anonymization, now better reflect underpinning core values at stake regarding, say, uses of participant data? If there has been a material change of circumstances, does more need to be done via the proxies of public engagement, openness, and accountability? For example, might a downstream participant engagement exercise help to test the values and tolerances in performances of trust relative to any proposed change in the research or deployment of new proxies?

- *3. Risks to proxies of trustworthiness*

This part of the framework moves actors’ analyses into a consideration of risk. This may assist in the identification of red flags that indicate that performances of trustworthiness are in jeopardy. Thinking prospectively helps anticipate risks and identify mitigating action. But what might risk look like?

There are three risk-based options that we might consider: (i) a crisis in the HHR endeavour; (ii) a material change in the HHR circumstances; and (iii) evidence of a breach of trust in the endeavour.

An illustrative example that encompasses all three triggers is a French clinical trial of a neurological drug that involved healthy volunteers (Feldwisch-Drentrup 2017). The research was catastrophic and left one participant dead and five others with brain damage (*The Guardian* 2016). Trust was breached here in at least two respects. First, when participants agreed to take part in the trial, they signed a consent form which stated: “You will be informed about any new significant information that could affect your willingness to continue the trial” (Enserink 2016). They were not, however, made aware that another volunteer had become seriously ill. Second, the company and its collaborators refused to publish pre-trial data after the trial collapsed to protect “industrial property.” These are clear examples of how a proxy of trustworthiness (consent) was not followed through as the research endeavour proceeded and how another proxy of trustworthiness (openness) was not adequately performed relative to its values (respect, autonomy, and care). Both failings illustrate what a red flag for trustworthiness might encompass. An ongoing audit of the validity of the proxy of trustworthiness of consent could have averted a risk to trust that resulted in collapse of the trial. Equally, a clearer commitment to openness could have mitigated any further damage to trust and the reputations of the company and collaborators.

### Trustworthiness: Is There a Cause for Concern?

After undertaking the analysis encouraged by these three steps, research actors will be in a stronger position to anticipate whether the proxies of trustworthiness under scrutiny are doing enough work to shore up the trustworthiness of their endeavours or whether any red flags have been raised. This is because the proxies in play and the underlying values that are being promoted (or not promoted) have now been identified and reflected upon critically. Once this analysis is undertaken, the framework suggests two courses of action.

#### Action 1: Continue with the HHR endeavour.

If working through the three steps leads the actor to conclude there is no concern regarding trustworthiness (as far as that it possible to assess), they are encouraged to continue with their endeavour, but nonetheless to revisit the cycle as the research protocol proceeds through the trajectory. This revisiting is important because risks to trust are ever-present and shift throughout the trajectory. A strong performance of trustworthiness at one point on the trajectory does not mean that trustworthiness is maintained throughout, and far less that other proxies deployed at other junctures will be assessed similarly.

#### Action 2: Re-evaluate the values and proxies and consider whether new proxies of trustworthiness are needed.

If the research actor concludes that there is valid concern about trustworthiness, they are urged to reconsider which values are in play and whether more could be done to strengthen existing proxies. As part of this exercise, actors should also consider whether new proxies of trustworthiness are needed to strengthen general and particular performances of trustworthiness. To return to the French example, early reflection could have led to better engagement and communication with participants about what had happened and what steps were needed to perform future trustworthiness well, including a change in direction of the research and more care paid to participants.

A further example comes from experiences of the COVID-19 pandemic. In the wake of the World Health Organization declaration of a pandemic on March 11, 2020, pharmaceutical companies mobilized internationally to bring vaccines to market. In response, regulators such as the U.S. Food and Drug Administration (FDA) and the European Medicines Agency (EMA) instituted regulatory reforms to expedite scientific review, to institute rolling reviews of data in parallel with the approvals process, and to reduce evaluation timeframes (EMA 2022a). As a further consequence of these rapid regulatory responses, a shift occurred from pre-authorization scrutiny driven by safety and efficacy to post-authorization pharmacovigilance, i.e., following the data about vaccine use in the population. While this is scientifically sound, it nonetheless raises ethical questions and possible concerns for public trust. Were governments’ economic imperatives to get citizens back to work overriding previous ethical imperatives to fully test safety and efficacy? Can pharmacovigilance mechanisms adequately protect populations and ensure sufficient protection of privacy, given that detailed scrutiny of patients’ data will be required for such a system to be effective? The dilemma is this: does the value of public interest in rapidly and effectively countering a pandemic carry sufficient weight to support these regulatory reforms when they might increase risks to individual citizens’ rights and interests? This is a global issue: consider, most recently, FDA proposals to test COVID boosters solely on mice and not humans as sufficient to bring to market (Stein 2022). If such expedited review measures are not trusted, then no amount of new vaccines will make any difference because mistrust will simply drive vaccine hesitancy.

Our purpose here, however, is not to discuss vaccine hesitancy (Dubé et al. 2015) but rather proxies of trustworthiness. In this example, the public interest imperative has driven rapid regulatory change. Established proxies of trustworthiness such as consent, anonymization, and public engagement are far less relevant in this new context. Previously in Europe, in the light of numerous pharmaceutical safety concerns, a mechanism had been established to allow citizens to participate in safety reviews (Altavilla 2018); but the highly truncated timelines involving COVID-19 suggest this measure is now far less feasible and effective. And while both the FDA (FDA 2022) and the EMA (EMA 2022b) have striven to be fully transparent about their motives and actions, this is not the same as openness which might require access to data for assessibility and explicability purposes, as noted above. Accountability remains to be seen. But, in this new climate, is there room for new proxies of trustworthiness to emerge? An important development in Europe was the COVID-19 EMA Pandemic Task Force, since superseded by the Emergency Task Force which is “… an advisory and support body that handles regulatory activities in preparation for and during a public-health emergency, such as a pandemic” (EMA 2022c). This body now has a legislative basis (Regulation EU 2022/123, Article 15). It is precisely such a body that could benefit from the framework advocated herein. Crucial to this are two features: first, its remit would need to be broadened beyond the scientific elements of a response to include the socio-ethical; and second, its composition would need to include members with bioethical expertise. Thus, while the question remains open as to which new proxies of trustworthiness might emerge in new regulatory landscape, there is hope that institutionally and structurally the formal elements are in place to approach the question lest these regulatory shifts do result in a crisis of trust. Indeed, we might even consider the Task Force itself as an *institutional* proxy of trustworthiness.^2^

In other contexts, other novel proxies of trustworthiness might be relevant. For example, it is well-documented that (most) publics are more trusting of public institutions than commercial enterprise conducting research. Here, a proxy of trustworthiness that might address concerns is benefit sharing, if not of specific profits, then certainly of data and new knowledge (Haddow et al. 2007). This would reflect the underlying value of solidarity. Health and social inequalities are also reflected in many areas of HHR, either because populations are (inadvertently) exploited or because they are excluded from participation or from just benefits arising from research (Selden and Berdahl 2020). Here, a new proxy of trustworthiness we might call “affirmative access” could begin to address such injustices, underpinned by the value of justice itself (Cash-Gibson et al. 2021).

Continuing with the core value of justice, London has argued most recently and convincingly that a commitment to the common good in HHR requires that an even wider network of actors have moral responsibilities for the proper conduct of research, including pharmaceutical companies, philanthropical organizations, affected communities, and even editors of journals (London 2022). Such an expanded moral community would also benefit from the proxies of trustworthiness framework and would contribute extensively to its refinement, the articulation and evaluation of proxies of trustworthiness, and the overall trustworthiness of HHR.

### The Role of Reflexivity in This Framework

Proxies of trustworthiness are uncertain beasts in the HHR ecosystem. This makes it inappropriate to speak of optimization of proxies of trustworthiness because this would set impossibly high standards and result in endless rounds of regulatory inefficiency. This must be avoided. Instead, the framework encourages processes of ongoing reflexivity in parallel with the conduct and review of research. The argument herein is fundamentally an ethical one: it embraces the fragility of trust in HHR and recognizes that attempts to perform trustworthiness are themselves built on sand. Reflexivity can be built into the system as part of ongoing training of researchers, regulators, ethics committees, funders, peer reviewers, etc. (Samuel et al. 2022).

### The Strengths of This Framework

We suggest that a strength of this framework is that it exhibits a further fundamental value: humility. It does not purport to have all the answers to how trustworthiness can be performed *well* in HHR. Rather it provides a guide to assist research actors to have a maximal chance of performing their trustworthiness relative to performances of trust upon which the entire research edifice is built. The use of values rather than rules to assist in this task offers flexibility that complements the fickle and fluid nature of trust. It might be easy to say “carry out some public engagement to demonstrate openness as a proxy of trustworthiness and elicit the value of respect,” but this would be little more than an exercise in ethical paint-by-numbers. A more subtle, nuanced, and open approach is needed to reflect the complexities of the HHR ecosystem and the various sociocultural contexts where HHR takes place—where different values may come into prominence at different times (Nortjé et al. 2021). This, in turn, means that proxies of trustworthiness also shift, as do the priorities of their underpinning values. It is for this reason that the framework consists of a system of feedback loops, promoting continuous commitment of the HHR ecosystem and its actors to improve through aspiring to strengthen performances of trustworthiness. Those who use this framework therefore become stewards of trustworthiness in HHR by contributing to its success as a learning system.

### The Limitations of This Framework

Some may harbour scepticism that any trustworthiness indicator can be manipulated or faked (Kramer 2009). But such reservations are precisely why this framework takes a values-based approach. It encourages reflection on how far values are aligned in performances of trustworthiness and trust. The imperative to seek ways to align values *through* performances of trust and trustworthiness makes faking trustworthiness much harder.

Concerns about fakery might raise a further criticism that this framework relies on research actors to assess their own performances of trustworthiness. Critics might suggest that a self-referential approach will inevitably involve bias. This concern can be addressed by the fact that the framework suggests each actor in the ecosystem should feed into, and return to, the framework throughout the course of their research to elucidate where more needs to be done to perform trustworthiness *well*. In other words, the framework itself requires a network of trust between actors at various stages of the research trajectory who can act as a check on each other. Further research will be needed to see how this works in practice, but the expectation is that actors’ assessments using the framework converge and coalesce in a fashion similar to the Delphi method, which has proven worth in promoting collective consensus on policy and practice issues (RAND 2023).

It might also be objected that a misalignment of values will lead automatically to a conclusion about a breakdown in trust or the presence of mistrust when this might not be so. But no such conclusion should be reached too quickly. A key proxy of trustworthiness—public engagement—could be deployed in such a scenario to test whether trust was indeed under threat. The framework prompts reflection and serves as a starting point to evaluate and assess proxies of trustworthiness. It does not replace the value of hard evidence about trust itself.

This brings us to a final possible criticism, viz., the development of this framework has not taken an empirical approach. Certainly, it does not look at the “state” of trust in HHR by referencing polls or other indicators of public opinion. But this is deliberate because the changeable nature of trust limits the value of empirical studies (O’Higgins et al. 2018). Indeed, it is these shifts in trust that give this framework durability and legitimacy. Empirical evidence does, indeed, have a role within the framework. For example, to support assessment of whether proxies of trustworthiness are—or are not—working well (for now). Also, empirical evidence can “test” the operation of the framework and its underlying premise, viz., that the protection and promotion of core values can indeed serve to engender trust. But this is not the same as saying that empirical evidence is central to the operation of the framework itself, and it certainly does not follow that an assessment of trustworthiness at a given moment in time says anything about whether trust will be forthcoming in the future.

## Conclusion

This paper offers an answer to the following question: *how can trust continually be promoted in an ever-changing and uncertain HHR environment?* It does so by proposing that trust and trustworthiness be seen as performative acts relative to each other and the common values at stake. It argues that research actors perform through *proxies of trustworthiness* and that this conceptualization should be adopted to better reflect and reveal the ways in which key mechanisms of protection and promotion of values within the HHR ecosystem operate when oriented towards gaining trust. The notion of a proxy of trustworthiness reflects the fragility and precarity of trust itself. Moreover, proxies of trustworthiness are best understood as an interconnected and interdependent network of mechanisms to perform trustworthiness relative to performances of trust and the values that are common to both. The paper offers a novel values-based framework to strengthen performances of trustworthiness across the HHR ecosystem and a mechanism by which existing and future proxies of trustworthiness can be identified, assessed, maintained, or replaced in rapidly changing HHR regulatory environments. By these means, the relationship between trust and trustworthiness in HHR is revealed at a deep theoretical level and research actors are equipped with a practical normative tool to better align performances of trustworthiness and trust relative to the values at stake in HHR.

## Footnotes

1 We borrow this approach from Xafis et al. 2019.

2 A similar example of a body being a proxy of trustworthiness is the creation of the U.K. Biobank Ethics and Governance Council which was charged to act as a critical friend to the researchers and to serve as a guardian of the Ethics and Governance Framework that was established to protect participants.
